# Supplementary material for: Metagenomic functional profiling: to sketch or not to sketch?
Source: Bioinformatics. 2024 Sep 4;40(Suppl 2):ii165–73. doi: 10.1093/bioinformatics/btae397 (PMC11373326; doi:10.1093/bioinformatics/btae397)
Supplement: btae397_Supplementary_Data [file btae397_supplementary_data.pdf]

Background

KEGG and gene orthology

Selecting an appropriate reference database is crucial in metagenomic functional profiling. Orthologous genes, which share a common evolutionary ancestry, are commonly employed for inferring functions (Fang *et al.*, 2010). Numerous efforts have been undertaken to construct databases for gene orthology, such as NCBI COG (Galperin *et al.*, 2015), AGNOSTOS-DB (Vanni *et al.*, 2021), OrthoDB (Kriventseva *et al.*, 2019), and Ensembl Compara (Herrero *et al.*, 2016). The KEGG database is a comprehensive and integrated resource for biological interpretation by providing gene annotations and mapping genes to manually created pathway maps (Kanehisa and Goto, 2000; Kanehisa *et al.*, 2017). These KO groups serve as a foundation for functional annotation in metagenomics and can be further connected into pathway maps to illustrate the hierarchical interconnections of biological functions within living organisms (Kanehisa *et al.*, 2016; Lesker *et al.*, 2020; Fritz *et al.*, 2019b).

FracMinHash and sketching-based methods

With a large number of reference genomes and an exponential surge in metagenomic data production, there is an imperative need for the creation of computational models that are both scalable and robust, ensuring precision in analysis. K-mer-based algorithms, particularly sketching-based methods, are gaining increasing popularity for metagenomic profiling implementations in this regard. A k-mer is a sequence of k consecutive nucleotides, extracted from a longer sequence. Algorithms designed to work with k-mers split the entire sample into k-mers, and analyze the number of shared/dissimilar k-mers among multiple samples. The number of all distinct k-mers in a sequencing sample can often be huge, so sketching-based methods take a fingerprint of the k-mers (called a *sketch*) and work with these much smaller sets, ensuring less consumption of computational resources. The most popular sketching method for many years has been MinHash, introduced in the context of document comparisons (Broder, 1997). Mash (Ondov *et al.*, 2016) was developed to apply MinHash to genomic data and has been very widely used. However, recent studies have shown that the error in comparing two MinHash sketches depends on the sketch size (Pătraşcu and Thorup, 2012), and the size needs to grow quadratically to compensate for the error (Ondov *et al.*, 2016, Fig S1). More recent works have shown that when sets of very dissimilar sizes are compared, MinHash sketches perform relatively poorly (Liu and Koslicki, 2022; Koslicki and Zabeti, 2019). Researchers have proposed many adjustments to MinHash to tackle this issue (Ondov *et al.*, 2019; Koslicki and Zabeti, 2019; Blanca *et al.*, 2022; Jain *et al.*, 2018); using a variable sketch size (instead of MinHash's fixed-size scheme), the recently introduced FracMinHash sketch (Irber Jr, 2020; Hera *et al.*, 2023) is one such example. Simply speaking, a FracMinHash sketch retains a fraction of the k-mers in the original sample. Formally, given a perfect hash function $h : \Omega \to [0, H]$ for some $H \in \mathbb{R}$ and a *scale factor* s where $0 \leq s \leq 1$, a FracMinHash sketch of a set A is defined as follows:

$$\mathbf{FRAC}_s(A) = \{\, h(a) \mid a \in A \text{ and } h(a) \leq Hs \,\}\,. \tag{2}$$

The scale factor s is a tunable parameter that can modify the size of the sketch. For a fixed s, if the set A grows larger, the sketch $\mathbf{FRAC}_s(A)$ grows proportionally. This sketching technique was first introduced in the software package `sourmash` (Brown and Irber, 2016; Pierce *et al.*, 2019), and has recently been theoretically analyzed to justify its use (Hera *et al.*, 2023). `sourmash` has successfully used FracMinHash to obtain genome-wide comparisons of biological sequences. In the second round of Critical Assessment of Metagenomic Interpretation (CAMI) challenges (Sczyrba *et al.*, 2017; Meyer *et al.*, 2022), the tool `sourmash gather` (Irber *et al.*, 2022), exhibited the highest completeness and purity in taxonomic profiling from metagenomic data for multiple datasets (Meyer *et al.*, 2022) across the genus and the species levels.

Definitions of metrics used

In this section, we describe the metrics we used to benchmark `fmh-funprofiler` and `DIAMOND`. We first give a high-level textual definition, and then give the rigorous mathematical formula. To write the mathematical formulas, we need to introduce a few notations first.

Let the set of KOs truly present in the ground truth be $G = \{K_{g_i}, 1 \leq i \leq m\}$, and the set of KOs predicted by a tool be $P = \{K_{p_j}, 1 \leq j \leq n\}$. Also, let the abundance of a KO K_{g_i} in the ground truth be $a_{g_i}, 0 < a_{g_i} \leq 1$, and the abundance of a KO K_{p_i} in the predicted output be $a_{p_i}, 0 < a_{p_i} \leq 1$. We also assume that the KOs in the ground truth are ordered based on the non-increasing order of abundances, i.e. for any $i \leq j$, $a_{g_i} \geq a_{g_j}$.

The metrics we used in our analyses are:

- Precision: the fraction of KOs that are truly present amongst the discovered KOs.

$$P(G, P) = \frac{|G \cap P|}{|P|}$$

- Completeness: the fraction of the gold standard KOs identified by the tools.

$$C(G, P) = \frac{|G \cap P|}{|G|}$$

- Completeness in top 95% KOs: the fraction of the top 95% abundant gold standard KOs identified by the tools. To mathematically define this, let $G_{95} = \{K_{g_i}, 1 \leq i \leq k, \text{where } k \text{ is the smallest integer that } \sum_{i=1}^{k} a_{g_i} \geq 0.95\}$. Then, this metric is as follows:

$$C(G_{95}, P) = \frac{|G_{95} \cap P|}{|G_{95}|}$$

- Weighted Jaccard similarity: weighted Jaccard index of the abundances of the KOs in the ground truth, and those identified by the tools. To define this, we define the weight functions w_G and w_P as follows: if $K \in G$, then $w_G(K)$ gives the abundance of K in ground truth, and 0 otherwise. Also, if $K \in P$, then $w_P(K)$ gives the abundance of K in the prediction, and 0 otherwise. Using these functions, weighted Jaccard similarity is defined as follows:

$$J_w(G, P) = \frac{\sum_{K \in G \cup P} \min\left(w_G(K), w_P(K)\right)}{\sum_{K \in G \cup P} \max\left(w_G(K), w_P(K)\right)}$$

- Correlation: the Pearson correlation coefficient of the abundances of the ground truth KOs, and those identified by the tools (only limited to the correctly identified KOs). Standard Pearson correlation was used, and therefore, we are not including the mathematical definition for this metric here.
- Bray-Curtis distance: the Bray-Curtis distance of the abundances of the ground truth KOs, and those identified by the tools (only limited to the correctly identified KOs). Bra-Curtis distance is often used to measure the dissimilarity in species composition between two samples. We used Bray-Curtis to quantify the dissimilarity in KO composition.

$$BC(G, P) = 1 - 2\frac{\sum_{K \in G \cup P} \min\Big(w_G(K), w_P(K)\Big)}{\sum_{K \in G \cup P}\Big(w_G(K) + w_P(K)\Big)}$$

Supplementary figure: functional landscape of the gut microbiota

Details of functional profiles of HMP data

We used command `sourmash sketch` with parameters `-p protein, k=11, abund, scaled=1000` for FracMinHash sketches of the HMP data (though it's suggested for the reference data to have multiple k values to fit various purposes, one proper k size is sufficient for functional profiles). Next, functional profiles of them were generated using `sourmash prefetch` with parameters `-k 11 --protein --threshold-bp 500` ($k = 11$, which corresponds to 33 in DNA sequences, is a reasonable value for protein sequence comparison).

Differential analysis

LEfSe (Linear discriminant analysis Effect Size) was performed to determine enrichment in functional profiles of pairs of conditions. We adhered to the default settings for this analysis. Features (KO or pathway) with LDA score (log10) higher than 2 and adjusted p-value < 0.05 were recognized as significant. We used the Seaborn package in Python for plotting. For simplicity, we only showed partial results in the manuscript.

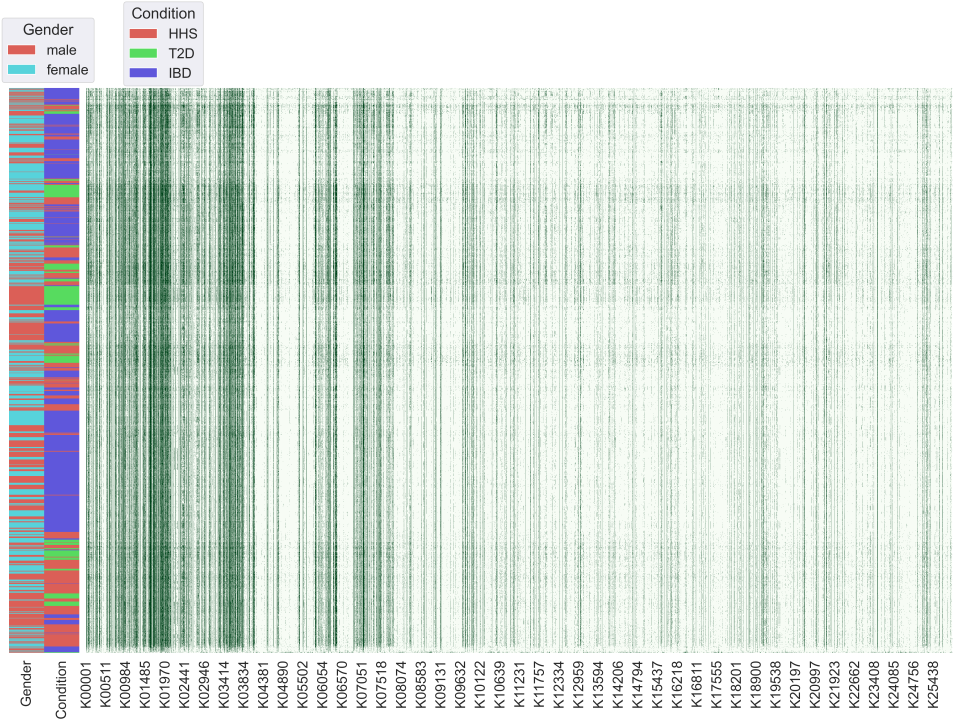

(a) Functional landscape of the gut microbiota.

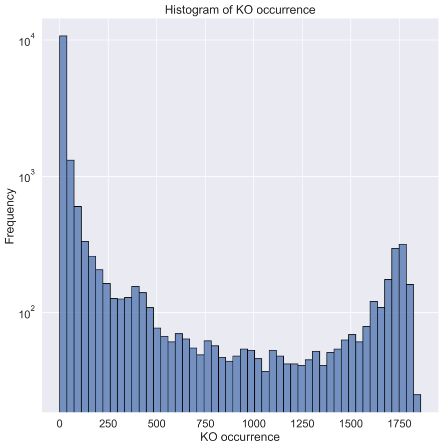

(b) Frequency of KOs detected in all samples

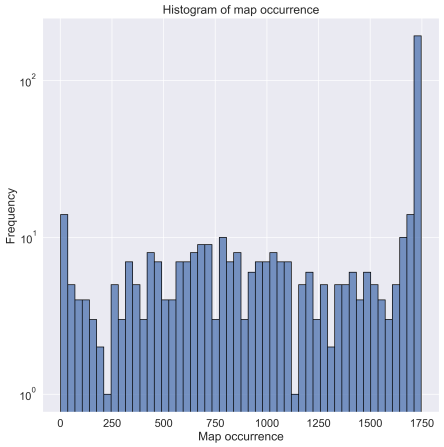

(c) Frequency of KEGG pathways detected in all samples

Fig. 5. Functional landscape of the gut microbiota. (a) Heatmap of KO occurrences in all gut metagenomic samples from HMP database. Each row represents one metagenomic sample and each column shows the presence of a given KO across all samples. Samples are clustered by KO profiles. A vertical green "line" suggests that a KO shows up in most of the samples. (b) Frequency distribution of KO occurrences: the right side contains KOs identified in most of the samples, i.e. "housekeeping". (c) Frequency distribution of KEGG pathway occurrence.
